# 1337 nm Emission of a Nd^3+^-Doped TZA Glass Random Laser

**DOI:** 10.3390/nano13131972

**Published:** 2023-06-29

**Authors:** Jessica Dipold, Camila D. S. Bordon, Evellyn S. Magalhães, Luciana R. P. Kassab, Ernesto Jimenez-Villar, Niklaus U. Wetter

**Affiliations:** 1 Instituto de Pesquisas Energéticas e Nucleares, IPEN/CNEN, Av. Prof. Lineu Prestes, 2242, São Paulo 05508-000, SP, Brazil; jessica.dipold@alumni.usp.br; 2 Departamento de Engenharia de Sistemas Eletrônicos, Escola Politécnica da USP, São Paulo 05508-220, SP, Brazil; camiladsb@usp.br (C.D.S.B.); evellynmagalhaes@gmail.com (E.S.M.); 3 Faculty of Engineering and Natural Sciences, Tampere University of Applied Sciences, 33100 Tampere, Finland; 4 Faculdade de Tecnologia de São Paulo, CEETEPS, São Paulo 01124-060, SP, Brazil; kassablm@fatecsp.br; 5 Instituto de Física “Gleb Wataghin”, Universidade Estadual de Campinas, Campinas 13083-859, SP, Brazil; ernesto.jimenez@uv.es; 6 CREOL, The College of Optics and Photonics, University of Central Florida, Orlando, FL 32816, USA

**Keywords:** random laser, scattering media, tellurite glass, neodymium-doped materials

## Abstract

Random lasers have been studied using many materials, but only a couple have used glass matrices. Here, we present a study of zinc tellurite and aluminum oxide doped with different percentages of neodymium oxide (4 wt.%, 8 wt.%, and 16 wt.%) and demonstrate for the first time random laser action at 1337 nm. Laser emission was verified and the laser pulse’s rise time and input–output power slope were obtained. A cavity composed of the sample’s pump surface and an effective mirror formed by a second, parallel layer at the gain-loss boundary was probably the main lasing mechanism of this random laser system. The reason for the absence of emission at 1064 nm is thought to be a measured temperature rise in the samples’ active volume.

## 1. Introduction

Letokhov proposed random lasers (RLs) theoretically for the first time in the 1960s, with their first experimental demonstration conducted in 1993 by Gouedard et al. [1,2]. These lasers differ from regular lasers in that the stimulated emission is caused by scattering between particles or differences in the refractive index within the sample [3], while, for normal lasers, a physical cavity is always responsible for the emission gain. For this reason, RLs are cheaper and easier to fabricate than regular lasers and have a wide range of applications, including remote sensing, encryption, and cancer detection.

Currently, RLs are fabricated from dyes [4], crystal powders [5], polymers [6], fibers [7], and nanoparticles doped with rare-earth ions [8], among other materials [9,10,11,12]. High-efficiency RLs have been demonstrated in neodymium (Nd)-doped materials [5], showing the promising capabilities of this kind of rare-earth random laser. Usually, proof of random laser action is evidenced through the observation of linewidth narrowing once it reaches the stimulated emission regime and by a slope increase in its power emission curve after the laser threshold. Another proof of random lasing is an abrupt decrease in the emission decay time from the spontaneous emission time down to the pumping pulse width. 

There is a lack of literature regarding glass for RL applications [13,14]. Glass is interesting for RL manufacturing because it can be highly doped with rare-earth ions, which grants the freedom to make selections from a wide range of emission wavelengths, mechanical stabilities, broad tuning capabilities, and generations of ultrashort pulses. This versatility makes glass a captivating choice for the production of RL devices, providing numerous possibilities for tailored and advanced applications. It has large mechanical resistance, can have a high refractive index, and is usually easier and cheaper to fabricate than crystals. 

However, studying random laser effects in glass can be very challenging [15] since the main proof for random laser action is linewidth narrowing, which does not occur in glass because of its inhomogeneous broadening [15]. Additionally, it has a lower damage threshold than crystal because of its large amount of dangling bonds and crystalline defects, which are especially present in the ground powders of random lasers, introducing an appreciable residual absorption. This makes it difficult to achieve laser threshold in glass powders since the lower pumping power necessary to avoid damage is offset by the lower emission cross-section of glass that would require higher pump powers to achieve the threshold. The latter, combined with the very low thermal conductivity of glass, makes random laser action in glass powders a challenge.

One possible method to verify random lasing in glass is through its temporal behavior. Measuring the emission decay time makes it possible to evaluate the transition from spontaneous to stimulated emission. If a second decay time that is shorter than the spontaneous emission time is observed during the measurement, this suggests the potential involvement of stimulated emission in the signal, even prior to reaching the threshold. It has been shown that laser threshold occurs when the participation of stimulated emission in the RL is 50% [16]. At even higher pump powers, stimulated emission eventually dominates, to the point that a short pulse appears at the beginning of the emission, and laser threshold is achieved. This random laser behavior expresses itself in a soft threshold transition, contrary to the abrupt threshold of bulk lasers. Such studies have been conducted previously for a crystal powder RL doped with Nd^3+^ and for dye solutions [16,17]. 

Another characteristic that can be obtained from the temporal measurements is the rise time of the signal. Shi et al. demonstrated that by observing the decrease in the rise time of a rhodamine dye, it is possible to find the lasing threshold in a similar way to that observed during traditional linewidth narrowing [18]. 

Usually, Nd-doped materials present stimulated emission only at 1064 nm (^4^F_3/2_ → ^4^I_11/2_), but in certain conditions, 1.3 µm emission (^4^F_3/2_ → ^4^I_13/2_) also happens [19]. Since the emission cross-section of the 1064 nm emission is always larger, it is necessary to suppress it to be able to effectively detect the 1.3 µm emission. Few glass materials doped with Nd^3+^ ions have presented stimulated emission at 1.3 µm [20], and, even so, all of them were excited by an 800 nm pump source. In a random laser, the emission at 1.3 μm is even more challenging once there are no easy means of suppressing the 1064 nm emission. A material similar to ours has been studied before [13], focusing on the 1064 nm emission using a monochromator, which only allowed this wavelength to reach the detector, and not verifying other possible emissions that could be happening simultaneously.

Here, we present a temporal study of a random laser made from the powder of Nd_2_O_3_-doped TeO_2_-ZnO-Al_2_O_3_ glass in three different concentrations (4 wt.%, 8 wt.%, and 16 wt.%). For this study, a pulsed 585 nm laser source was used as the pump beam and, contrary to the expected 1064 nm emission, a 1337 nm stimulated emission was observed. After the threshold pump power was reached for each concentration, we verified a ~5 ns pulse overlapped on fluorescence emission, showing random laser behavior. By analyzing the intensity curve of this ~5 ns pulse as well as the rise time curve, we demonstrate the threshold for laser activity, showing for the first time, to our knowledge, a 1337 nm emission for a glass random laser.

## 2. Materials and Methods

The glass was prepared using the melt-quenching method, adding 4, 8, and 16 wt.% of Nd_2_O_3_ to the following starting composition: 85.0 TeO_2_-12.95 ZnO-2.05 Al_2_O_3_ (TZA). The melting of the reagents was conducted at 835 °C for samples with 4 and 8 wt.% of Nd_2_O_3_ and 1000 °C for those with 16 wt.% of Nd_2_O_3_. In the latter case, the high temperature was used to solubilize the high concentration of Nd_2_O_3_. Melting was conducted over 30 min using a platinum crucible of high purity (99.999%), followed by quenching in pre-heated brass molds and annealing at 300 °C for 120 min to reduce internal stress and make the samples less fragile. Afterwards, cooling down to room temperature was performed. The characterization of TZA glass (with a refractive index of approximately 2.0) was reported previously for 0.5 wt.% of Nd_2_O_3_ doping [21].

**Figure 1:**
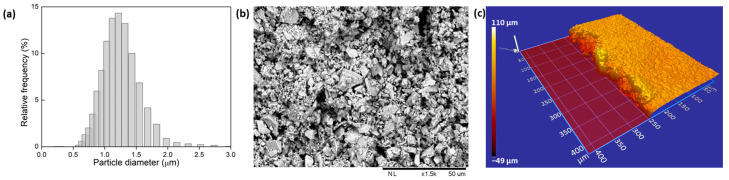
(**a**) Particle size distribution obtained through DLS measurements of the supernatant particles. (**b**) SEM image of the prepared sample surface. (**c**) Height map of the studied sample.

For each Nd_2_O_3_ concentration, the TZA glass was ground into powder by using an agate pestle. A sieve with a 20 µm mesh size was used in order to guarantee that only particles smaller than 20 µm were obtained for the sample preparation. Then, isopropyl alcohol was added and the mixture was placed in an ultrasound bath for three minutes to separate the supernatant particles. Particle hydrodynamic diameter was measured on four different powder samples, applying 25 measurements for each case, using dynamic light scattering (DLS; Litesizer™ 500 from Anton Paar, Graz, Austria); an average size of 1.5 µm was verified, as shown in Figure 1a. Through SEM imaging, we verified that the powders were polydisperse in size (Figure 1b), which was purposefully chosen due to previously published results [5], which demonstrated higher efficiency under these conditions.

A total of 100 mg of particles was selected and 0.1 mL of isopropyl alcohol was added in order to prepare a paste, which was then used to cover a 5 mm × 50 mm area on top of a microscope slide and left to dry at ambient temperature, forming a thin film with (56 ± 3) µm thickness. Three different slides were prepared, one for each Nd_2_O_3_ concentration (4 wt.%, 8 wt.%, and 16 wt.%). A scanning electron microscopy (SEM; HITACHI model TM3000, Tokyo, Japan) image of the sample with 8 wt.% of Nd_2_O_3_ is shown in Figure 1b, and a height map (Zygo ZeGage 3D optical surface profiler, Middlefield, OH, USA) of a small area of the same sample is shown in Figure 1c. 

**Figure 2:**
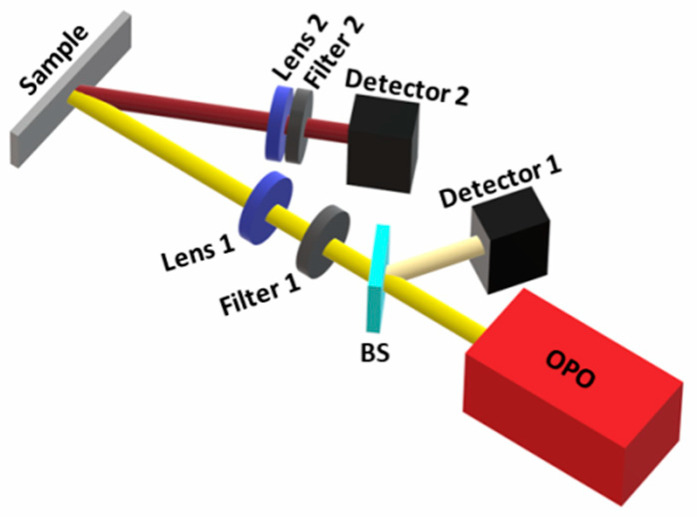
Experimental setup schematic. It shows the pump laser (OPO), which was partially reflected by a beam splitter (BS) of ~2% reflectivity into a reference detector (Detector 1), which was used as a trigger for the measurements; a short-pass filter of 600 nm (Filter 1) was used to let only the 585 nm pump wavelength pass; a 15 cm focal length lens (Lens 1) was used, which focalized the beam on the sample located on a xyz manual translator; a 2.5 cm focal length lens (Lens 2) was used to focalize the emitted beam into a photodetector (Detector 2); a filter, which was either a long-pass of 1200 nm to detect only 1300 nm or a bandpass for the detection of 1064 nm along with a long-pass filter of 1000 nm (Filter 2) was responsible for blocking the reflection of the 585 nm pump.

Temporal measurements were made for each slide using an OPO laser system (OPOTek model OPOlette, Carlsbad, CA, USA) with a pulse duration of 10 ns at a repetition rate of 20 Hz emitting at 585 nm. A 4 GSa/S and 350 MHz bandwidth oscilloscope (RIGOL model DS4034) was used to verify the decay and rise time of the studied samples measured with a fast InGaAs detector (Thorlabs model DET01CFC, Newton, MA, USA). A schematic of the experimental setup can be observed in Figure 2. For the rise time of the emitted signal [18], a Python code was developed to measure the time interval between the minimum and maximum of the pulse’s leading edge because the oscilloscope’s built-in functions gave unreliable and unstable results. The same detector was used to measure output power for input pulse fluences between 0.25 mJ/mm^2^ and 3.44 mJ/mm^2^ by calculating the average of four measurements of the maximum of the pulse’s leading edge after subtracting the fluorescence for each power.

**Figure 3:**
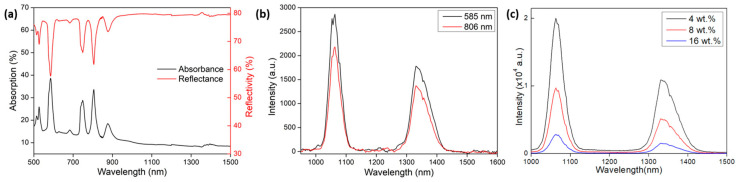
Absorbance, reflectance (**a**), and emission (**b**) for the 8 wt.% Nd_2_O_3_ sample; (**c**) fluorescence emission comparison for different neodymium doping concentrations.

Absorption and reflection measurements of the samples were taken using a Cary 5000 spectrometer (Figure 3a). For the reflectivity and absorption measurements, an integrating sphere was used in order to fully capture the diffusively scattered light. Spectral emission measurements were made by using the OPO laser at both 585 nm and 806 nm (Figure 3b), and the light was collected with a 200 µm fiber and using an OceanOptics^®^ NIRQuest spectrometer (Rochester, NY, USA) to observe the emission in the infra-red region.

## 3. Results and Discussion

### 3.1. Absorption and Emission Measurements

The absorbance and reflectance measurements (Cary 5000, Santa Clara, CA, USA, with integrating sphere) of the powder sample, excluding specular reflectance, are shown in Figure 3a. The most highly absorbing feature was the absorption peak at 585 nm, related to the ^4^I_9/2_ → ^4^G_5/2_ and ^2^G_7/2_ transitions of neodymium, which was the pump wavelength used throughout our experiments. To confirm the effectiveness of this pump wavelength, emission measurements were taken by using the OPO at 585 nm and the traditional pump wavelength of 806 nm (Figure 3b), collecting light with a 200 µm fiber and using an OceanOptics^®^ NIRQuest spectrometer (Rochester, NY, USA, 23 nm resolution) to observe the infra-red region. The same pump energy (0.7 mJ) was used for both wavelengths and the alignment was finely adjusted to maximize the signal in both cases.

As observed in Figure 3b, the emission strength achieved for the 585 nm pump wavelength was 31% higher than that for 806 nm, which is commonly used for Nd-doped laser pumping. In Figure 3c, a strong concentration quenching is observed, showing a lower signal strength for higher Nd_2_O_3_ doping, which, in return, gave a lower emission cross-section for the 16 wt.% Nd_2_O_3_ sample.

### 3.2. Rise Time Measurements

Measurements for both 1064 nm and 1337 nm emissions were taken by changing the filters used in front of the photodetector (see Figure 2). First, we analyzed the 1064 nm emission. To guarantee that during these measurements only 1064 nm was received by the detector, a long-pass filter of 1000 nm was used to block the pump wavelength at 585 nm and a second filter was used to block 98.9% of the 1337 nm wavelength (47.4% transmission at 1064 nm). For measuring the 1337 nm emission, long-pass filters of 1000 nm and 1200 nm were used, blocking all 1064 nm emissions. To test the effectiveness of the filter combinations, an Al_2_O_3_ diffusor was placed at the sample position, and the OPO was tuned to both 1064 nm and 1337 nm. The first setup leaked approximately 1.1% of the 1300 nm signal, while the second setup showed no 1064 nm emission.

**Figure 4:**
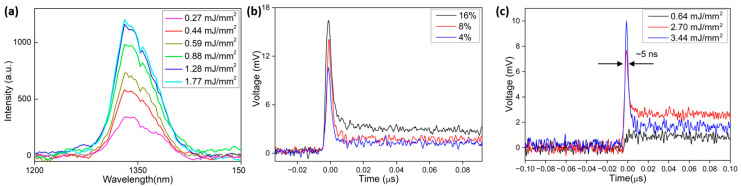
(**a**) Random laser emission at 1337 nm, showing no variation of the FWHM, as expected from glass. (**b**) Random laser pulses at 1337 nm measured for the 4 wt.%, 8 wt.%, and 16 wt.% Nd_2_O_3_ samples at 1.4 mJ pump energy (3.44 mJ/mm^2^). (**c**) Signal before threshold (0.64 mJ/mm^2^ pump power), slightly above threshold (2.70 mJ/mm^2^ pump power), and far above the threshold (3.44 mJ/mm^2^) for the 8 wt.% Nd_2_O_3_ doped sample.

Emissions for different pump fluences before and after laser threshold at 1337 nm can be observed in Figure 4a for the 8 wt.% Nd_2_O_3_ concentration, demonstrating that no linewidth narrowing could be detected.

At high pump powers, the 1064 nm fluorescence emission showed a second, shorter decay time, but without reaching the laser threshold. The 1337 nm emission showed a clear laser threshold for all three Nd_2_O_3_ concentrations (Figure 4b), and laser pulses were observed, as shown in Figure 4c for the 8 wt.% Nd_2_O_3_. The duration of this short pulse was approximately 5 ns, measuring the FWHM of the short peak as shown in Figure 4c.

**Figure 5:**
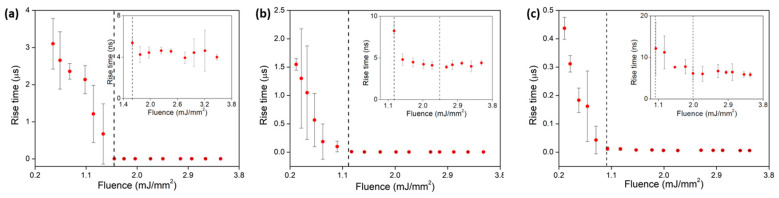
Rise time measurements for the 1337 nm emission for (**a**) 4 wt.%, (**b**) 8 wt.%, and (**c**) 16 wt.% Nd_2_O_3_ samples, with insets showing a zoom of the stationary part in nanoseconds scale.

The decrease in rise time as a function of pump power showed a clear threshold, after which a constant time was obtained (Figure 5). The point where the decreasing slope transitioned to the constant value was considered the lasing threshold for this observation method, as shown in [18]. The samples doped with 4 wt.% Nd_2_O_3_ decreased initially from 3.1 µs down to 4 ns (total difference of 3.096 µs). For the sample of 8 wt.% Nd_2_O_3_ doping, the decrease was from 1.55 µs to 4 ns, and for 16 wt.% Nd_2_O_3_, it was from 0.44 µs to 6 ns. For the 16 wt.% sample, we observed two different decay times: one from 0.1 mJ/mm^2^ to 1.0 mJ/mm^2^ and another from 1.0 mJ/mm^2^ to 2.0 mJ/mm^2^ (inset of Figure 5c), from where it remained constant.

The threshold for laser operation was (1.59 ± 0.02) mJ/mm^2^ for the 4 wt.% Nd_2_O_3_ sample, (1.10 ± 0.10) mJ/mm^2^ for the 8 wt.% sample, and (1.00 ± 0.02) mJ/mm^2^ for the 16 wt.% sample. The latter was contradictory since the emission cross-section measured at 1337 nm decreased with increasing Nd_2_O_3_ doping (Figure 3c). The odd behavior described above may have been caused by the formation of an effective cavity whose edges were formed by the input sample surface and the interface between coherent and incoherent emissions at a depth approximately corresponding to the average transport length within the pumped sample, as discussed in detail in Section 3.4.

### 3.3. Emitted Peak Power Measurements

**Figure 6:**
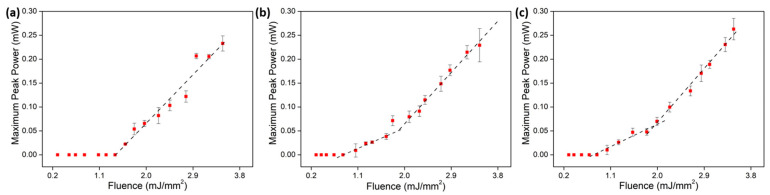
Maximum peak power per fluence for TZA samples doped with (**a**) 4 wt.%, (**b**) 8 wt.%, and (**c**) 16 wt.% of Nd_2_O_3_.

The emitted maximum peak power as a function of time was acquired for each pump power. Since we originally measured the maximum peak voltage (*V*) for each fluence, a calculation of the maximum peak power was performed by using the photodetector (Thorlabs DET01CFC) responsivity and the resistance (*R*) used in the oscilloscope (50 Ω) through a simple formula (W=V/(Responsivity×R). The laser’s peak height was measured by subtracting a baseline which was obtained by scaling the fluorescence signal below the threshold (fluence of approximately 0.5 mJ/mm^2^) to the fluorescence in the tail of the peak. The average of four measurements of the peak’s height, taken at four different positions, is shown in Figure 6, plotted separately for each Nd_2_O_3_ concentration.

The lasing threshold for each Nd_2_O_3_ concentration was (1.4 ± 0.4) mJ/mm^2^, (0.95 ± 0.10) mJ/mm^2^, and (0.81 ± 0.10) mJ/mm^2^ for the 4 wt.%, 8 wt.%, and 16 wt.% samples, respectively, which followed the trend observed for the thresholds obtained by the rise time measurements. The slope calculated for the 4 wt.% sample was (5.1 ± 0.5) mV·mm^2^/mJ and showed a monotonically increasing curve. For the higher concentrations, however, we observed two different slopes as the pump fluence increased. For the 8 wt.% curve, the initial threshold was 0.95 mJ/mm^2^ with a slope of (3.3 ± 0.5) mV·mm^2^/mJ and a second threshold at 2 mJ/mm^2^ with a slope of (5.4 ± 0.3) mV·mm^2^/mJ. The 16 wt.% sample had an initial threshold at 0.81 mJ/mm^2^ with a slope of (2.6 ± 0.4) mV·mm^2^/mJ and a second threshold at 1.8 mJ/mm^2^ with a slope of (5.9 ± 0.3) mV·mm^2^/mJ. 

### 3.4. Effective Cavity Demonstration

The fact that there is a progressive slope efficiency increase for higher doped samples has been observed before in different types of random lasers, such as polymer and glass RLs, and has been attributed to a transition from coherent to incoherent lasing [6,22]. The effect was attributed to long-range fluctuations of refractive indexes in the polymer films, most likely caused by the inhomogeneity of the film thickness [22]. Similar behavior has also been observed in [23] and was theoretically treated as a traditional DFB (distributed feedback) laser in the under-coupling regime. Feedback would be generated mainly by scatterers near the border of the pump volume at a depth of *l_a_* (absorption length) because shorter cavities would present a lower Q-factor and longer cavities would suffer reabsorption. 

Coherent emission due to an effective cavity induced by a layer composed of the input sample surface and the interface between gain and reabsorption inside the pumped sample was unequivocally linked to the spectral emission of a rhodamine-doped aerogel random laser [10]. This laser system, characterized as weakly scattering (*l_s_* > *l_a_*) with an *l_a_* of 53 µm and a scattering length (*l_s_*) of approximately 6 mm, demonstrated that the free spectral range (FSR) of the coherent emission peaks occurring near the laser threshold corresponded to a cavity length approximately 25% longer than the absorption length *l_a_*. When the pump power was further increased, incoherent emission (ASE, extended modes) took over and a single broad peak was detected. The transition from coherent to incoherent emission as a function of pump power depended strongly on the sample’s pump absorption, and the stronger the absorption, the stronger the coherent emission and the higher the necessary pump power to observe ASE. The maximum measured output power and the almost complete suppression of ASE up to the highest pump powers occurred for the shortest measured cavity length that coincided with the shortest absorption lengths [10]. 

In the present embodiment of a cavity-enhanced random laser, the system operated in the strongly scattering regime (*l_a_* > *l_s_*) with approximately the same absorption length as above of *l_a_* = 65 ± 1 µm for the 16 wt.%, measured in bulk samples with a spectrometer (Agilent, Santa Clara, CA, USA, model Cary 5000), and a much smaller transport mean free path of *l_T_* = 4.11 ± 0.9 µm [5], as determined by measuring the average width of the backscattering cone of all samples. For the complete characterization of the samples, the filling fraction was estimated from the transmission values of the samples. 

Knowing that 2.54% of the pump light was transmitted through the 16 wt.% Nd_2_O_3_ powder sample, we could estimate the macroscopic absorption length (*l_ma_*):

(1) $$\frac{I}{I_{0}}=e^{\frac{-d_{0}}{l_{ma}}}\to l_{ma}=-\frac{d_{0}}{ln(I/I_{0})}=-\frac{56}{\ln\left(0.0254\right)}=15.4\;\mu m$$

where *I* is the intensity of the light after passing through the sample, *I*_0_ is the intensity of the light before passing through the sample, and *d*_0_ is the total thickness of the powder layer, given by the height map of the sample of Figure 1c, which was on average 56 µm. The filling fraction (*ff*) was given by the following equation:

(2) $$ff=\frac{l_{a}*l_{T}}{3*l_{ma}^{2}}=38\%$$

This value was within the expected range once this was not a closely packed system that has a filling fraction of approximately 60%. 

It can be assumed that the pump depth at which positive gain (inversion) is achieved is considerably shorter than the absorption length since a high threshold was observed for these lasers (see Figure 6). To corroborate this further, the random laser was pumped through the glass-sample interface (back), where the light reflection coefficient should be lower than at the air-sample interface given the index matching between glass and sample. 

**Figure 7:**
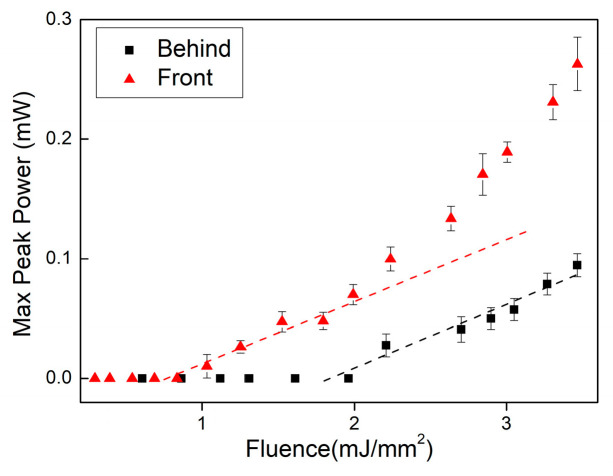
Maximum peak power per pump fluence for the 16 wt.% Nd_2_O_3_ sample for directly pumping the powder surface from the front (triangles) and for pumping from behind through the glass slide (bullets).

As shown in Figure 7, pumping the sample through the glass slide resulted in a similar slope as pumping from the front directly into the sample, but at a much higher emission threshold (two times higher) that was coincident with the second threshold observed above in Figure 6c and attributed to the contribution of extended modes. This is strong evidence of the above-explained cavity effect, which could be observed only from the sample’s front. In addition, the random laser emission collected from the side of the sample (parallel to the sample surface) was several times less than that for the frontal collection. The latter showed that the effect could not be attributed to ASE and also helped to confirm the hypothesis of the laser cavity mechanism in this random laser system in a resonator that involved the sample’s pump surface.

### 3.5. Oscillation at 1337 nm

**Figure 8:**
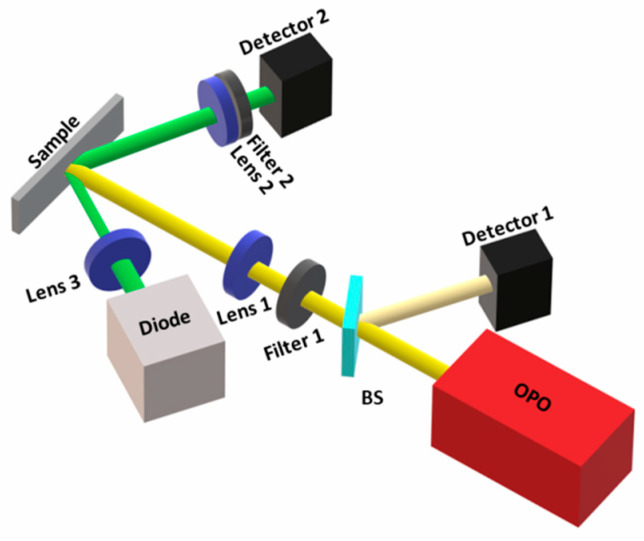
Setup to measure temperature variation. OPO, Filter 1, Lens 1, and Detector 1 were the same as in the previous setup. A diode laser of 532 nm was focused with a 50 mm focal length lens (Lens 3) within the 585 nm laser focus on top of the sample. The scattered light was collected by a 25 mm focal length lens (Lens 2), with a short-pass 550 nm filter (Filter 2) to block the 585 nm scattered light and allow only 532 nm into a Newport fast detector (Detector 2).

**Figure 9:**
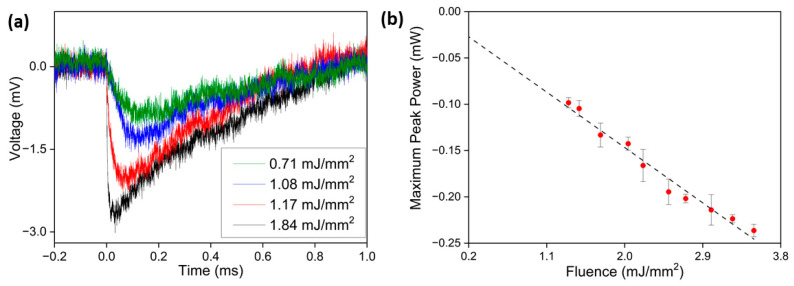
(**a**) Graphic of 532 nm diode scattering signals for different fluencies and (**b**) intensity measurements for different fluences for the 8 wt% Nd_2_O_3_ sample, showing linear behavior.

Since we observed laser action at 1337 nm but not at 1064 nm, even though this should be the stronger emission given the spectra of Figure 3, we studied possible reasons for this odd behavior of TZA:Nd. One possibility was the decrease of the available inversion population for the ^4^F_3/2_ → ^4^I_11/2_ transition (1064 nm) due to the thermal population of the lower laser level, situated approximately 2000 cm^−1^ above the ground level ^4^I_9/2_. The high concentration of glass defects, especially after being ground into powders, greatly enhances the probability of lower laser level population because they can cause intermediate energy levels between ^4^I_9/2_ and ^4^I_11/2_. Pump fluences higher than 2 mJ/mm^2^ caused visible burned spots on the samples (possible stoichiometric changes caused by segregation of the components), indicating that temperatures were higher than the segregation temperature (around the melting temperature—835 °C). For the highest fluences (3 mJ/mm^2^), the increase in temperature was enough to cause the ejection of the laser material, indicating that the temperature caused by the pump laser irradiation was well above the melting temperature. To study this phenomenon, a setup was prepared (Figure 8), where we focused (spot size 0.3 mm) a 532 nm CW laser diode into the same focal spot on the sample as the 585 nm OPO pulse (spot size 0.6 mm). The 532 nm diode had 53.4 mW output power and was placed at 45° with respect to the OPO pump beam. The heating of the sample surface caused by the 585 nm pulse induced a decrease in the reflected light (532 nm) at the sample-air interface. This 532 nm scattered light from the sample was focused with a 25 mm lens into a fast Newport Si detector. The oscilloscope trace plotted as a function of 585 nm pump fluence is shown in Figure 9a for the 8 wt.% sample, showing a strong decrease in the reflected signal as pumping fluence is increased.

Also visible was a faster decay time for the higher pulse fluences (above 1.08 mJ/mm^2^). This change in decay times was approximately coincident with the laser threshold. In Figure 9b, the peak signal changes are plotted as a function of pump fluence for the 8 wt.% Nd_2_O_3_ sample, demonstrating a linear change with pump power, which allowed us to infer a possible linear increase in the sample’s surface temperature. Notice that the surface temperature should increase proportionally with the energy absorbed by the sample. Therefore, a linear decay behavior of the reflected intensity indicated that the sample surface temperature increased linearly with the changes in the signal peak in Figure 9a. For pump fluences above 0.71 mJ/mm^2^, we observed burn spots of brown coloration after each pump pulse (each pulse occurring at a different location, as explained above), which were possibly due to the segregation of components of lower vapor pressure (temperature approximately 835 °C), which would lead to stoichiometric changes in the sample (coloration). For fluences higher than 0.90 mJ/mm^2^, a hole was formed due to the extraction of the material. The components’ segregation by the temperature increase occurred on a time scale much longer than that for the laser pulse and did not affect the measured emission intensity significantly during pump pulse duration, as shown by the linear increase of the slopes in Figure 6; nevertheless, it was clearly present, as shown by Figure 9a. Only if more than one pump pulse was focused onto the same location would a strong decrease in emission power be detected for each following pulse. 

The temperature required to reach a thermal population of 1% in the ^4^I_11/2_ energy level was 350 °C and, from the above, it can be concluded that at least some parts in the region of the focal spot reached the melting temperature at fluences between 0.71 and 0.9 mJ/mm^2^, i.e., below the threshold, and reached temperatures that could be more than three times as high at the highest pump fluences after the passage of the pump pulse (see Figure 9b). A detailed inspection of the curve with a 1.17 mJ/mm^2^ pump fluence in Figure 9a shows that the signal dropped by 20% of its maximum value during the first 150 nanoseconds and by more than 30% for the curve with 1.84 mJ/mm^2^. Additionally, although this would mean a small temperature increase of no more than 20 °C in the sample’s volume responsible for the deflection of the 532 nm probe laser, the temperature increase was probably considerably higher in the small active layer responsible for the laser action, which was of the order of several micrometers, as explained above (*l_ma_*= 15.4 μm and *l_T_* = 4.1 μm). This would indicate that inversion is strongly decreased by reabsorption (1064 nm), even during the short duration of the pump pulse.

Previous studies showed that different neodymium transitions behave differently with temperature increases [24,25] and, in most cases, the 1064 nm emission decreases at a higher pace than the 1300 nm transition. All studies that describe the neodymium emission variations at 1064 nm or 1300 nm as a function of temperature [24,26,27] used an 808 nm laser as the pump source. Sato et al. measured the 1064 nm emission intensity as a function of temperature for Nd:YVO_4_, amongst other crystals, and measured a 22% decrease for a temperature elevation from 20 °C to 65 °C and calculated an emission intensity decrease of more than 70% at a temperature of 300 °C [27]. Additionally, the 1337 nm emission peak, measured in Figure 3b, was 67% the height of the 1064 nm peak, which was comparably more than six times higher than in, for example, Nd:YAG. These are plausible reasons for the existence of the 1337 nm radiation.

## 4. Conclusions

Random laser emission was verified in TZA glass samples doped with three different concentrations of Nd_2_O_3_ (4 wt.%, 8 wt.%, and 16 wt.%) through rise time and output power measurements. The slope of the output power showed a clear threshold that coincided within 15% with the change in slope of the rise time measurements. The highest threshold at (1.4 ± 0.4) mJ/mm^2^ occurred for the lowest Nd_2_O_3_ concentration (4 wt.%) and the smallest threshold was found for the sample with 16 wt.% Nd_2_O_3_ at (0.81 ± 0.10) mJ/mm^2^. 

Emissions at 1337 nm were observed for the first time in a random laser, to our knowledge, whereas no laser emissions at 1064 nm occurred, which was attributed to strong sample heating. We attribute the 1337 nm emission to the increase in temperature of the thin, active laser sample layer, which favored the electronic population of the ^4^I_11/2_ energy level (lower laser level for 1064 nm emission), hampering inversion, stimulating emission at 1064 nm, and favoring the laser emission at 1337 nm.

A strong decrease in laser intensity was measured when pumping the sample from the back through the glass slide and when measuring parallel to the sample surface, which was attributed to a reflection loss at the index-matched glass-sample interface and cavity geometry, respectively. These effects corroborate our interpretation of a coherent laser emission originating inside the cavity formed by the sample surface and the gain-loss boundary that occurred inside the sample. This mechanism was pronounced and became stronger the more the transport mean free path approached the macroscopic absorption length, which, in our case, happened as absorption increased, i.e., for the higher wt.% Nd_2_O_3_ sample.

## Data Availability

The data presented in this study are available upon request from the corresponding author.
